# Response to Letter to the Editor From S. I. Taylor et al: “Type B Insulin Resistance Syndrome: A Rare Cause of Hypoglycemia”

**DOI:** 10.1210/jcemcr/luae002

**Published:** 2024-02-01

**Authors:** Salman Zahoor Bhat, Sooyoung Lim, Aniket Sidhaye, Amir H Hamrahian

**Affiliations:** Division of Endocrinology, Diabetes and Metabolism, John Hopkins University School of Medicine, Baltimore, MD 21287, USA; Division of Endocrinology, Diabetes and Metabolism, John Hopkins University School of Medicine, Baltimore, MD 21287, USA; Division of Endocrinology, Diabetes and Metabolism, John Hopkins University School of Medicine, Baltimore, MD 21287, USA; Division of Endocrinology, Diabetes and Metabolism, John Hopkins University School of Medicine, Baltimore, MD 21287, USA

**Keywords:** autoimmune hypoglycemia, type B insulin resistance syndrome, anti-insulin receptor antibody, diabetes mellitus, pancreatic cyst

Dear Editor,

We welcome the response of Taylor et al to our paper [1]. We appreciate their detailed insights on the pathophysiology of hypoglycemia in type B insulin resistance syndrome (TBIRS). We agree with the authors that our patient presents an atypical case regarding her C-peptide level. As the authors have correctly indicated, in most cases of patients with hypoglycemia and TBIRS, the C-peptides are typically low. We thank the authors for pointing out the error in Table 2 and the Discussion section.

The elevated C-peptide in TBIRS has also been noted in a few other case reports. Kang et al (2013) noted an elevated C-peptide of 1.7 nmol/L with a blood glucose of 18 mg/dL on presentation and a C-peptide of 1.6 nmol/L with a blood glucose of 32 mg/dL during a diagnostic fasting test in their case report [2]. Viswanathan et al reported a case of hypoglycemia in TBIRS with weakly positive antibodies, with an elevated C-peptide level of 9.1 ng/mL and a concurrent glucose level of 49 mg/dL [3].

As the authors have suggested, elevated C-peptide level in our patient may be due to a delay in achieving a new steady state of C-peptide levels in the hypoglycemic period. Alternatively, another complicating factor in our case is that our patient oscillated between hypoglycemic and hyperglycemic states. We suggest that although low C-peptide levels may be typical in TBIRS as suggested by Taylor and colleagues, the possibility of a nonsuppressed C-peptide level should be kept in mind while evaluating hypoglycemic patients with suspicion of TBIRS.

Thank you

Salman Zahoor Bhat

Sooyoung Lim

Aniket Sidhaye

Amir H. Hamrahian

## Data Availability

Data sharing is not applicable to this article as no datasets were generated or analyzed during the current study.
